# Impact of COVID-19 on the Hong Kong Youth Quitline Service and Quitting Behaviors of Its Users

**DOI:** 10.3390/ijerph17228397

**Published:** 2020-11-13

**Authors:** Laurie Long Kwan Ho, William Ho Cheung Li, Ankie Tan Cheung, Wei Xia, Man Ping Wang, Derek Yee Tak Cheung, Tai Hing Lam

**Affiliations:** 1 School of Nursing, University of Hong Kong, Hong Kong, China; longkwan@hku.hk (L.L.K.H.); tankie@hku.hk (A.T.C.); xiavive@hku.hk (W.X.); mpwang@hku.hk (M.P.W.); derekcheung@hku.hk (D.Y.T.C.); 2 School of Public Health, University of Hong Kong, Hong Kong, China; hrmrlth@hku.hk

**Keywords:** COVID-19, tobacco use, young smoker, youth health

## Abstract

Tobacco use is a possible risk factor for contracting and spreading COVID-19. We aimed to describe the impact of the COVID-19 pandemic on the Youth Quitline service and quitting behaviors of its users in Hong Kong. We conducted a telephone survey involving 201 participants of the Youth Quitline service, and retrospectively analyzed the operation and use of Quitline since the COVID-19 outbreak in Hong Kong. The number of incoming calls to the Youth Quitline and the participants′ quit rate has increased since the COVID-19 outbreak in Hong Kong. Many participants (68%) did not realize that tobacco use potentially increased their risk for developing and spreading COVID-19; however, 43% agreed that the pandemic motivated their intention to quit, and 83% changed their smoking habits during the pandemic. These changes were mainly due to wearing masks (30%), closure of bars/pubs (25%), suspension of classes (14%), and being unable to socialize with friends (24%). Overall, 58% reduced their tobacco use; of these participants, 66% reported a ≥50% reduction in daily cigarette consumption. The participants reduced their smoking during the COVID-19 pandemic despite lacking knowledge about the potentially increased risk for contracting COVID-19 from continued smoking. The pandemic could create new opportunities to motivate young smokers to quit smoking, especially those seeking support for smoking cessation, and may further contribute to reducing the risks posed by COVID-19.

## 1. Introduction

The coronavirus disease 2019 (COVID-19) pandemic expanded rapidly across the globe, with more than 44 million confirmed cases and 1.1 million deaths reported in October 2020. The World Health Organization (WHO) indicated that tobacco use is a major risk factor for contracting COVID-19 because of its harmful effects on the lungs and possible viral transmission from fingers to mouth during smoking [1,2]. However, considerable controversy exists regarding the relationship between smoking and COVID-19 [3,4]. Nevertheless, both tobacco use and COVID-19 can cause damage to the cardiovascular and respiratory systems [1]. Smokers infected with COVID-19, particularly those who already have tobacco-induced diseases, are at higher risk for developing severe COVID-19 symptoms and death than non-smokers [1,4,5]. Given the potential risks of tobacco use, the pandemic could provide new opportunities for promoting smoking cessation to reduce the health hazards from smoking and COVID-19.

Governments worldwide have adopted strict measures to combat the spread of COVID-19. Following the first confirmed COVID-19 case in Hong Kong, reported on 23 January 2020, the Hong Kong government announced various measures including social distancing and other regulations that significantly affected people′s everyday lives [6]. The impact of the COVID-19 pandemic on people′s daily activities, routine habits, and everyday practices remains underexplored. In particular, little is known about young smokers′ quitting, smoking behavior, and the tobacco cessation services for youth due to the disruptions caused by the pandemic. Public awareness about the relationship between COVID-19 and smoking is crucial for promoting smoking cessation and reducing the health hazards during the pandemic. Currently, only limited evidence exists on the risk perceptions of continued smoking during the pandemic among young smokers. Understanding their risk perceptions could provide information for healthcare professionals, which may facilitate the design of appropriate interventions for promoting smoking cessation among young smokers during the pandemic.

As the most developed city in China, Hong Kong has the lowest smoking prevalence (10.2%) in the developed world and a low youth smoking prevalence (2.5%) [7,8]. To promote smoking cessation among young people, the Smoking Cessation Research Team (from the School of Nursing and School of Public Health of the Li Ka Shing Faculty of Medicine, Department of Social Work and Social Administration, University of Hong Kong) and the Hong Kong Council on Smoking and Health established the world′s first peer-led toll-free smoking cessation hotline in Hong Kong, called the Youth Quitline (YQ), in 2005 [9]. The YQ is recognized as a core partner of the WHO Collaborating Centre for Smoking Cessation and Treatment of Tobacco Dependence. Details of the YQ service have been described elsewhere [10]. Briefly, the YQ adopts a non-pharmacological approach, using trained peer counselors to receive telephone inquiries, provide smoking cessation counseling to young smokers aged ≤25 years, and follow-up on their quitting progress. Outreach programs are also structured for schools and other educational institutions by having booths set up to entice more young smokers to use the hotline. The hotline is reported to have a satisfactory quit rate of 23.6% [10]. The present study aimed to describe: (1) the impact of COVID-19 on the YQ users’ quitting behavior, (2) their risk perceptions of smoking during the pandemic, and (3) the impact of COVID-19 on the YQ service.

## 2. Materials and Methods

We conducted a cross-sectional telephone survey from 24 April to 8 May 2020 with a YQ cohort of young smokers. Ethical approval was obtained from the Institutional Review Board of the University of Hong Kong/Hospital Authority Hong Kong West Cluster (Ref: UW 05-185 T/848). We included YQ participants who were: (1) aged ≤25 years; (2) current smokers who had consumed cigarettes in the previous 30 days; and (3) Cantonese speakers. We identified potential eligible participants from current YQ users and contacted them to ask for their willingness to participate in the telephone survey. Eligible participants who verbally consented to be interviewed were asked to respond to a standardized and structured questionnaire in a telephone interview. The questions developed by a group of tobacco cessation experts covered smoking behavior, readiness to quit, and risk perceptions of smoking during the pandemic. Trained peer counselors conducted the interviews using a computer-aided telephone system. The interviews lasted for 5–15 min and were audiotaped.

To enhance understanding about the situation of young smokers during the pandemic, the trained peer counselors recorded notes of their verbal communication as additional qualitative data when participants shared their opinions or feelings. A research assistant also transcribed verbatim the audio records to ensure data integrity. Thematic analysis was used to analyze the qualitative data [11]. Two researchers independently read through the transcript several times to ascertain a general sense of the data. Codes were generated by collating relevant data pertaining to the research question. Important quotes relevant to the study were identified and translated into English. Any divergence of opinion was managed during research team meetings. In addition, we retrospectively analyzed the operation and use of YQ since the outbreak of COVID-19 in Hong Kong (from February 2020 to April 2020).

## 3. Results

**Figure 1:**
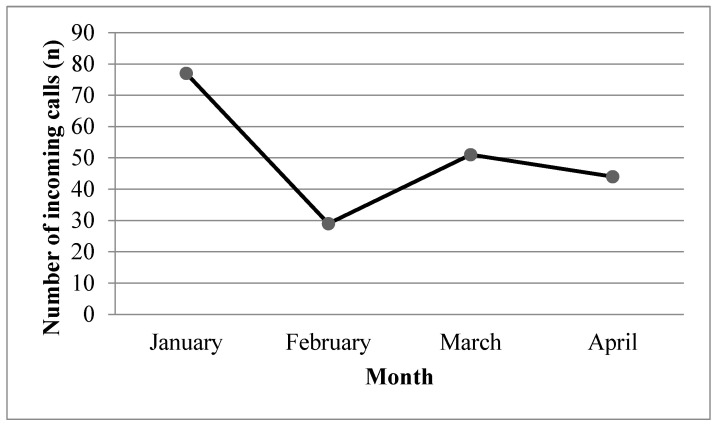
Number of incoming calls to Youth Quitline by month ^a^. ^a^ Repeated calls from the same caller were counted as one incoming call.

**Figure 2:**
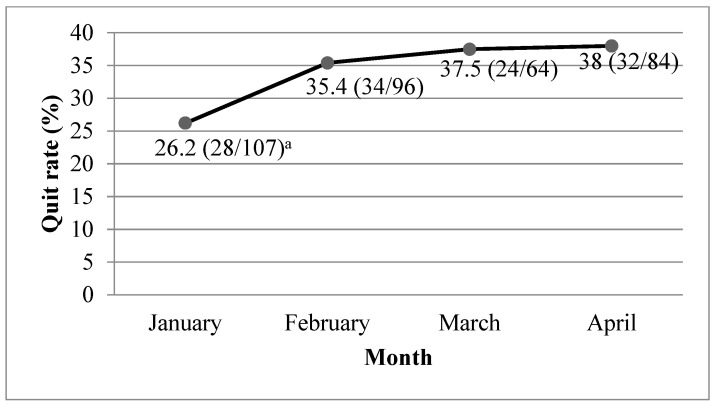
Quit rate among Youth Quitline participants by month. ^a^ Numerator = number of participants whose abstinence was biochemically validated; denominator = number of participants who completed follow-up.

All YQ outreach programs and promotion activities had to be suspended from February 2020 because of the closure of schools. This resulted in a significant decline in the number of incoming calls to the YQ. However, the number of incoming calls increased by 43% in March, as many young smokers called to seek help with quitting smoking because of the unfavorable environment for smoking (Figure 1). Due to the suspension of all face-to-face YQ services during the pandemic, we conducted smoking cessation biochemical validations through online video calls. A video demonstrating the procedure and a test strip were sent to the participants. Our staff monitored the whole procedure via video calls and provided necessary support for the participants throughout the procedure. The quit rate, which is defined as biochemically validated abstinence from tobacco for the past 7 days, increased from 26.2% in January 2020 to 38% in April 2020 (Figure 2). We also determined that 2% of YQ participants were unable to have regular follow-ups because the border closures that aimed to reduce imported cases meant they were detained in China or other countries and could not return to Hong Kong.

Of the 369 current YQ users, 311 were eligible to participate in this study and were contacted. Of these, 201 completed the telephone survey, 45 refused to participate, and 108 were not reachable after six attempts. The response rate was 65% of all eligible participants (201/311) and 82% of all reachable participants (201/246). Most participants were male (82%) and the mean age was 19 years (standard deviation = 2.7 years).

### 3.1. Risk Perceptions of Smoking Related to COVID-19

Table 1 shows the responses of participants to the close-ended items in the questionnaire. Of the participants, 68% did not think that tobacco use would increase their risk of developing COVID-19. In addition, 61% disagreed that tobacco use would increase their risk of spreading COVID-19 to their families and friends.

### 3.2. Impact of COVID-19 on Quitting and Smoking Behavior

A total of 43% of participants agreed that the pandemic motivated their intention to quit and 76% stated that their smoking habits had been changed or affected by the pandemic (Table 1). The changes were mainly because of wearing a mask (30%), closure of bars and pubs (25%), suspension of classes (14%), and being unable to socialize with friends (24%). In addition, 58% of participants reported a reduction in tobacco use; of these, 66% reported a ≥50% reduction in daily cigarette consumption.

During the telephone interviews, 54 participants (27%) shared their situation in relation to how the pandemic and corresponding control measures had affected their smoking habits. Examples of statements are presented in Table 2. The participants stated that class suspension was a major reason for not allowing them to smoke. This was mainly because they were required to stay home, and most of their parents were not aware of their smoking habits. In addition, the social distancing regulation also prohibited them to have gatherings with friends. Some of the participants expressed their thoughts about quitting due to the unfavorable environment for smoking and lack of peer pressure. They also mentioned that wearing masks made it more difficult to smoke in public areas. However, many participants felt doubtful about the increased risk of smoking in spreading and developing COVID-19. Most of them were not aware that smoking might be a possible factor for developing COVID-19.

## 4. Discussion

This study explored the impact of the COVID-19 pandemic on quitting and smoking behavior among a population of young smokers seeking smoking cessation support. We also assessed their awareness of the smoking hazards during the pandemic. In this study, a majority of young smokers did not realize that tobacco use was a possible risk factor for developing and spreading COVID-19, which suggests that more health education should be provided to enhance their risk awareness of smoking during the pandemic. Tobacco control advocates should use this golden opportunity to promote smoking cessation, which may further reduce the risks from COVID-19.

The findings indicated that the increased anti-epidemic measures since the outbreak peak in March 2020 significantly changed the everyday lives of young smokers, and put them in a position to think about quitting. We found that nearly half of the participants were motivated to quit due to the pandemic. A majority had also changed their smoking habits, reporting a reduction in daily cigarette consumption. These results supported the results for Youth Quitline, which showed that both the number of incoming calls and quit rate increased since the COVID-19 outbreak in Hong Kong. Schools were shut in late January 2020. Students therefore needed to learn in the confinement of their own homes without face-to-face interactions with friends. To prevent community clusters of infections, the Hong Kong government ordered the closure of cinemas, karaoke lounges, bars, and pubs in March. Together with a ban on gatherings of more than four people in public areas and other social distancing rules, all these measures prevented young smokers from gathering in these venues. Since young smokers were accustomed to smoking with their friends [12], they had less opportunity to smoke during the pandemic. Some also admitted that they had hidden their smoking habits from their parents, and that smoking at home was not allowed [13]. In addition, Hong Kong residents are adamant that they have had almost 100% mass masking in the community since the outbreak. The findings showed that widespread use of face masks in Hong Kong meant that smoking was inconvenient for young smokers.

The findings showed that the change in smoking behavior of many YQ participants during the pandemic may increase their intention to quit smoking in the long term. The pandemic could provide new opportunities to promote smoking cessation among this population, and potentially in other age groups, as most people (smokers and non-smokers) would have increased awareness and concerns about disease and death, and take precautions (such as mask wearing and social distancing) to protect their health. However, the accessibility of smoking cessation services during the pandemic was greatly reduced due to the suspension of face-to-face smoking cessation services. Healthcare institutions have also redeployed resources to handle the care of pandemic cases. Smoking cessation services using hotlines may be the only safe and accessible medium to continue the provision of counseling and advice for smokers. To maximize such opportunities to help smokers quit and in anticipation that the pandemic may continue for many months, we recommend that hotline services along with the use of information and communication technologies (e.g., instant messaging and social media) should be expanded when face-to-face services are limited. Mobile health intervention is widely used for health promotion and treatment compliance [14], which could provide a promising means to reinforce the smoking cessation service during the pandemic.

Although the YQ was able to sustain smoking cessation services during the pandemic when stringent social distancing regulations were implemented, its operation was affected. Specific strategies should be used to adjust the services according to the local situation and stage of the pandemic. First, the YQ operation hours were extended (from 5–9 p.m. to 9 a.m.–9 p.m.) to allow for more incoming calls, since young smokers may have more free time to call or receive counseling due to class suspension. Second, we enriched the content of smoking cessation counseling by providing information on risks associated with continued smoking during the pandemic: for example, water pipe tobacco, which is becoming popular in young smokers, can increase the risk of COVID-19 transmission [1], and the act of smoking can transmit the virus from fingers to mouth [1]. We offered advice based on risk communication, which was effective for promoting smoking cessation by discussing the risks associated with continued smoking [15]. Suggestions on how to maintain hand hygiene and stay healthy during the pandemic were also provided. We aimed to support the young smokers with useful information for maintaining a healthy lifestyle and staying aware during the COVID-19 pandemic. The operation of the YQ can provide a reference for other countries and regions to promote smoking cessation during the pandemic.

Increasing evidence shows that two-thirds of smokers die prematurely, especially those who started smoking at a young age [16,17,18]. Young smokers are also more likely to continue smoking in adulthood [19]. Smoking has profound negative effects on the healthy development of the young population. In addition to the risk of the pandemic, young smokers might be even more susceptible to the detrimental effects of smoking, such as increased susceptibility for respiratory infections and a weakened immune system. This study indicated that young smokers seeking smoking cessation support were changing their smoking behavior to adapt to the unfavorable environment and disruptions caused by the COVID-19 pandemic, which led them to think about quitting. This can be a great opportunity to promote smoking cessation among this population, which may further protect them from the risk of smoking and COVID-19. Nevertheless, statistics in the U.K. suggest that cigarette consumption or smoking relapse may increase during the pandemic [20]. It is thus imperative for healthcare professionals to advocate smoking cessation during the COVID-19 pandemic to ensure public health, especially for the young population.

This study had several limitations. First, the generalizability of our findings might be limited as we only included YQ participants as YQ is the only youth-targeted smoking cessation hotline in Hong Kong. Second, the young smokers who used the Quitline might have had greater motivation to quit and be more vulnerable to the impact of the pandemic than those who did not use the Quitline. Third, the effectiveness of providing information about the potential risks of smoking during the pandemic to promote smoking cessation remains unclear. It is recommended that further randomized controlled trials be conducted to examine the effectiveness of such a strategy.

## 5. Conclusions

Given the increased awareness and community actions around health protection and the benefits of quitting smoking in the current pandemic situation, tobacco control advocates should use this opportunity to promote smoking cessation. Strategies used to promote smoking cessation should be adjusted according to the local situation and stage of the pandemic. The Youth Quitline in Hong Kong can provide a reference for other countries and regions to promote smoking cessation among young smokers during the pandemic, especially for those who are seeking smoking cessation support. The pandemic could create new opportunities to motivate smokers to quit smoking, which may further reduce the risks from COVID-19.

## Figures and Tables

**Table 1:** Young smokers’ quitting and smoking behavior, and their risk perceptions of smoking during the pandemic (N = 201).

Questions	Responses, n (%)	
	Yes	No
Risk perceptions of smoking during the pandemic		
1. Do you think tobacco use will increase the risk of developing COVID-19?	64 (32)	137 (68)
2. Do you think tobacco use will increase the risk of spreading COVID-19 to your family or friends (e.g., secondhand smoke)?	78 (39)	123 (61)
Impact of COVID-19 on quitting and smoking behavior		
1. Has the pandemic increased your intention to quit?	86 (43)	115 (57)
2. Has the pandemic and the anti-epidemic measures affected your smoking behavior?	153 (76)	48 (24)
	***Main reasons (can choose more than one):*** - Wearing mask: 30% - Closure of bars and pubs: 25% - Suspension of classes: 14% - Unable to hang out with friends: 24% - Did not specify: 7%	
3. Has your daily cigarette consumption reduced during the pandemic?	116 (58) <50% reduction: 34% ≥50% reduction: 66%	85 (42)

**Table 2:** A summary of statements.

Examples of Statements
“I usually smoke with friends after school…We are unable to smoke since our school closed and [we] can’t hang out together.”
“My mom does not need to work because her boss asked her to have no-pay leave, which means that I need to stay at home with her every day. She would kill me if she realized that I have a smoking habit.”
“My parents want me to stay home and do not allow me to go out with friends. They think that it will increase the risk of developing the virus… I really miss the school days… smoke with friends make me feel relaxed.”
“I feel inconvenienced by pulling down the mask to smoke…People surrounding also look at me if I pull down my mask. It seems like I am infected and spreading the virus. I do not think the smoke can spread the virus, how ridiculous!”
“I have thought about quitting because I can’t smoke with friends now. I usually smoke when they ask me to because they won’t be friends with me if I reject them.”
“I don’t think smoking can spread COVID-19. It is a fallacy.”
“In my understanding, COVID-19 is transmitted by touching the things and by saliva…something like that… It seems not related to air or even related to smoking. I think it may be some misinformation on the internet.”
“I have been thinking of quitting for a while, but it is too difficult for me to do so. All my friends smoke, when we go out, we must smoke. However, I have not met my friends for few months. It seems to be a good chance for me to quit.”
“We have not needed to go to school since class suspension. I have no excuse to go out and smoke with my friends… My dad does not know that I smoke. He would talk to my class teacher if he realized this.”
“It becomes a common practice for me or for everyone to wear mask when go out. This reduces my desire to smoke in the public area because I do not want to pull down my mask.”
“Actually I do not have much money to buy cigarettes and my mom does not know I have a smoking habit. However, my friends and I usually go to the roof… We play and talk and smoke… My friends do not mind sharing cigarettes with me… But now we cannot go to school and cannot go out… I feel sad and hope everything will be fine soon.”
